# Range Verification Methods in Particle Therapy: Underlying Physics and Monte Carlo Modeling

**DOI:** 10.3389/fonc.2015.00150

**Published:** 2015-07-07

**Authors:** Aafke Christine Kraan

**Affiliations:** ^1^Department of Physics, National Institute for Nuclear Physics (INFN), University of Pisa, Pisa, Italy

**Keywords:** hadron interactions, Monte Carlo modeling, range verification, PET, prompt gamma

## Abstract

Hadron therapy allows for highly conformal dose distributions and better sparing of organs-at-risk, thanks to the characteristic dose deposition as function of depth. However, the quality of hadron therapy treatments is closely connected with the ability to predict and achieve a given beam range in the patient. Currently, uncertainties in particle range lead to the employment of safety margins, at the expense of treatment quality. Much research in particle therapy is therefore aimed at developing methods to verify the particle range in patients. Non-invasive *in vivo* monitoring of the particle range can be performed by detecting secondary radiation, emitted from the patient as a result of nuclear interactions of charged hadrons with tissue, including *β*^+^ emitters, prompt photons, and charged fragments. The correctness of the dose delivery can be verified by comparing measured and pre-calculated distributions of the secondary particles. The reliability of Monte Carlo (MC) predictions is a key issue. Correctly modeling the production of secondaries is a non-trivial task, because it involves nuclear physics interactions at energies, where no rigorous theories exist to describe them. The goal of this review is to provide a comprehensive overview of various aspects in modeling the physics processes for range verification with secondary particles produced in proton, carbon, and heavier ion irradiation. We discuss electromagnetic and nuclear interactions of charged hadrons in matter, which is followed by a summary of some widely used MC codes in hadron therapy. Then, we describe selected examples of how these codes have been validated and used in three range verification techniques: PET, prompt gamma, and charged particle detection. We include research studies and clinically applied methods. For each of the techniques, we point out advantages and disadvantages, as well as clinical challenges still to be addressed, focusing on MC simulation aspects.

## 1 Introduction

The main challenge in radiotherapy for cancer treatment is how to deliver high dose to the tumor region, while minimizing the irradiation of healthy tissue. One of the most important new modalities being developed for cancer therapy is irradiation with charged ions. Thanks to the characteristic dose deposition profile (Bragg peak), charged particles offer the possibility to deposit dose much more locally than the photons, so dose in healthy tissue can be minimized (1, 2). However, treatments with charged particles are more sensitive to uncertainties than photon treatments, because of their steep dose profile. Error sources include anatomical changes (e.g., organ motion, tumor regression, weight loss), patient setup errors and range errors from uncertainties in CT Hounsfield units (HU), conversion of HU into particle stopping power, and reconstruction artifacts (3). These can result in under-dosage to the tumor and unwanted dose to healthy tissue. Because of these uncertainties, in particle therapy clinics, generally large safety margins around the tumor are employed, and/or probabilistic or robustly optimized conservative treatment plans are used. This may not be optimal for the patient and may impair the beneficial effects of charged particle therapy. Much research in particle therapy is therefore aimed at developing new methods, which enable to verify the particle range in patients.

Various techniques for particle range verification have been developed over the last decades (4). Non-invasive *in vivo* treatment monitoring can be performed by detecting secondary particles produced as a result of nuclear interactions of the incident particle beam with the patient tissue, like *β*^+^ emitters, prompt photons, and charged fragments. Monte Carlo (MC) simulations have played a crucial role in the development and clinical application of range verification techniques. They can accurately describe particle transport and interactions of radiation with matter in complex geometries, such as fully detailed CT descriptions of the patient anatomy. This makes them a suitable tool for feasibility and detector design studies. Furthermore, *in vivo* non-invasive range monitoring methods generally rely on direct comparisons between measured and MC predicted distributions of secondary particles. The accuracy of the MC codes is therefore a crucial issue. Unfortunately, modeling nuclear interactions and the resulting secondary particle production is a highly complex task, because it involves nuclear physics interactions, for which no rigorous models exist.

Although much literature is available on interactions of charged particles in matter, reviews dedicated to particle therapy are scarce and nuclear interactions are often only discussed superficially. For instance, a dedicated review about interactions of charged particles in radiation therapy is written by Lomax (5), but it only very briefly touches on nuclear interactions. Moreover, it does not include range verification methods and MC models. The same applies to valuable reviews about physics of heavy charged particles (6, 7). Also, Gottschalk has written an excellent summary about proton interactions, including some nuclear physics (8), but it does not include MC codes and *in vivo* range verification. Very recently, a valuable review written by Newhauser and Zhang (9) about proton physics was published, including Monte Carlo and analytical modeling of proton interactions; however, nuclear interactions are discussed shortly and range verification techniques were not reviewed. And vice versa, thorough reviews about range verification methods are available (4), but the physics interactions and MC codes modeling them are described only very shortly. Extensive reviews exist about the usage of MC techniques in particle therapy (10, 11), but these do not contain a systematic description of interactions of charged hadrons in matter. Furthermore, the number of particle treatment centers around the world is growing, and thereby the demand for *in vivo* non-invasive range verification methods increases. In view of the rapidly evolving technical developments in the last years, we believe that an up-to-date description of the different range verification strategies, the state-of-the-art MC codes, and their underlying physics principles is timely.

This review intends to give a comprehensive overview of various aspects in modeling the physics processes that are relevant in range verification methods based on secondary particle detection in proton, carbon, and heavier ion irradiation. We will cover the physics principles behind the various range verification methods, the MC codes to simulate them, and the validation of the codes, including both clinically implemented methods as well as research studies. Hereby, we highlight the difficulties, limitations, and challenges related to physics modeling for range monitoring. This review is organized in the following way:

Section 2 is devoted to a brief description of interactions of charged particles in matter for energy ranges relevant in radiotherapy. We discuss both electromagnetic and nuclear interactions, and point out some practical consequences regarding beam fluence and dose. Moreover, we discuss some general approaches in modeling nuclear interactions, adopted by most state-of-the-art MC codes used in hadron therapy, and show how nuclear interactions give rise to production of *β*^+^ emitters, prompt *γ*’s, and charged fragments.

Range verification methods rely heavily on the accuracy of the particle transport code for describing dose deposition and nuclear fragmentation. Section 3 presents a summary of the available MC codes that are most widely used in particle therapy, and in particular in research related to particle range verification. For each MC generator, we briefly discuss how the relevant physics processes are modeled, and we give some examples of how these models are validated for proton and heavy ion therapy.

In Section 4, we review the use of MC codes in non-invasive particle range verification, focusing on three techniques: PET, prompt *γ*, and charged particle imaging. For each strategy, we describe selected examples of the application of the codes to treatment monitoring, as well as the available detectors. We also highlight some remaining clinical challenges regarding physics modeling.

In Section 5, we compare the three techniques, pointing out their strength and drawbacks. We also briefly touch on the development of hybrid systems. Finally, we describe some common efforts, which could improve the accuracy of signal prediction in treatment monitoring techniques.

## 2 Interactions of Charged Particles in Matter

In this section, we review electromagnetic and nuclear interactions of charged ions in matter. We narrow our focus to particle types and energies currently used in particle therapy centers worldwide. This means that we consider interactions of protons up to about 250 MeV and carbon ions up to about 450 MeV/u, i.e., penetrating into the human body up to about 40 cm. Before going into detail, let’s first quickly look at their typical velocities. For a particle of kinetic energy *E*_kin_, total energy *E*_tot_, mass *m*_0_, and momentum *p*, the particle velocity *β* in units of the velocity of light *c* is given by:

(1) $$\beta \equiv \frac{v}{c}=\frac{\mathrm{pc}}{E_{\text{tot}}}=\frac{\sqrt{E_{\text{tot}}^{2}-m_{0}^{2}c^{4}}}{E_{\text{kin}}+m_{0}c^{2}}=\frac{\sqrt{E_{\text{kin}}^{2}+2E_{\text{kin}}m_{0}c^{2}}}{E_{\text{kin}}+m_{0}c^{2}}$$

For a proton with kinetic energy *E*_kin_ = 250 MeV and given the proton mass = 938 MeV/c^2^, we find *β* ≈ 0.6, while a carbon ion with energy 450 MeV/u has *β* ≈ 0.7. Thus, in radiotherapy, we generally deal with moderately relativistic particles.

### 2.1 Electromagnetic interactions

#### 2.1.1 Electromagnetic Energy Losses for Charged Particles

Moderately relativistic charged particles interact with material by electrical (Coulomb) forces with the atomic electrons and with the material nuclei. The particle looses energy primarily by inelastic collisions with the atomic electrons, resulting in ionization and atomic excitation. These are *continuous* energy losses. When the ejected electron is so energetic that it can cause ionization itself, we call it a delta-ray. The amount of energy lost due to Coulomb interactions with the material nuclei is very small (12).

For charged particles other than electrons with charge number *Z*_p_ moving in a target material of atomic number *Z*_t_ and density *ρ* with velocity *β* larger than the orbital electron velocity, the mean ionization energy loss (or electronic stopping power) can be described by the Bethe-Bloch equation (12–14):

(2) $$\frac{\mathrm{dE}}{\mathrm{dx}}=K\rho \frac{Z_{\text{p}}^{2}}{\beta^{2}}\frac{Z_{\text{t}}}{A_{\text{t}}}\left[\frac{1}{2}\ln\left(\frac{2m_{\text{e}}c^{2}\beta^{2}\gamma^{2}T_{\text{max}}}{I_{\text{e}}^{2}}\right)-\beta^{2}-\frac{\delta }{2}-\frac{C}{Z_{\text{t}}}\right]$$

with $K=4\pi N_{\text{A}}r_{\text{e}}^{2}m_{\text{e}}c^{2}$, *N*_A_ Avogadro’s number, *r*_e_ and *m*_e_ are the radius and mass of the electron, *A*_t_ the molar mass of the material, $\gamma =\frac{1}{\sqrt{1-1/\beta^{2}}}$, *I*_e_ is the mean ionization potential of the material. Furthermore, *δ* is the density correction, relevant only for ultra-relativistic charged particles, and *C* is a shell correction term, which becomes important when the particle velocity becomes closer to the velocity of the atomic electrons. Heavy ions, which are fully stripped at high velocities, get partly neutralized by picking up electrons from the target material as they slow down. This decreases the particles’ effective charge (*Z*_p_)*_eff_* that has to replace *Z*_p_ in Eq. 2. The latter represents only the main contributions to the stopping power. There exist several higher-order corrections in *Z*_p_, which have been proposed to improve Eq. 2, like Barkas, Bloch, and Mott corrections. For a more extensive discussion, we refer to a comprehensive review by Ziegler (12). The ionization potential can be parameterized for instance in Ref. (15):

(3) $$I_{\text{e}}\left(Z_{\text{t}}\right)=\left(12Z_{\text{t}}+7\right)\;\;\;\text{eV for}Z_{\text{t}}\leq 13$$

(4) $$I_{\text{e}}\left(Z_{\text{t}}\right)=\left(9.76Z_{\text{t}}+58.8Z_{\text{t}}^{-0.19}\right)\;\text{eV for}Z_{\text{t}}>13$$

Here, *T*_max_ is the maximum kinetic energy, which can be transferred to a free electron in a single collision and is given, for an incident particle of mass *M*, in Ref. (14):

(5) $$T_{\text{max}}=\frac{2m_{\text{e}}c^{2}\beta^{2}\gamma^{2}}{1+2\gamma m_{\text{e}}/M+{\left(m_{\text{e}}/M\right)}^{2}}$$

For very low energies, when *β* becomes comparable or less than the velocity of the orbital electrons, the so-called Lindhard region, Eq. 2, is no longer valid. Then, the energy loss becomes proportional to *β* (16) and is of the order of:

(6) $$\frac{\mathrm{dE}}{\mathrm{dx}}≅8\pi \rho \frac{N_{\text{A}}}{A_{\text{t}}}\frac{\hbar^{2}}{m_{\text{e}}}\frac{Z_{\text{p}}^{7/6}Z_{\text{t}}}{Z}\frac{\beta }{\beta_{\text{o}}},$$

where $Z^{\frac{2}{3}}\;=Z_{\text{p}}^{\frac{2}{3}}+Z_{\text{t}}^{\frac{2}{3}}$ and $\beta_{o}=\frac{e^{2}}{4\pi \varepsilon_{0}\mathrm{\hbar c}}$ (≈0.0073) are the electron velocity in the classical lowest Bohr orbit of the hydrogen atom. In between the Bethe-Bloch and the Lindhard region, energy losses can be described by the low energy model of Anderson and Ziegler (17); alternatively, a polynomial can be used to join up the regions. For compound materials, the stopping power is the weighted sum of all the single elements, corrected for ionization energy.

**Figure 1:**
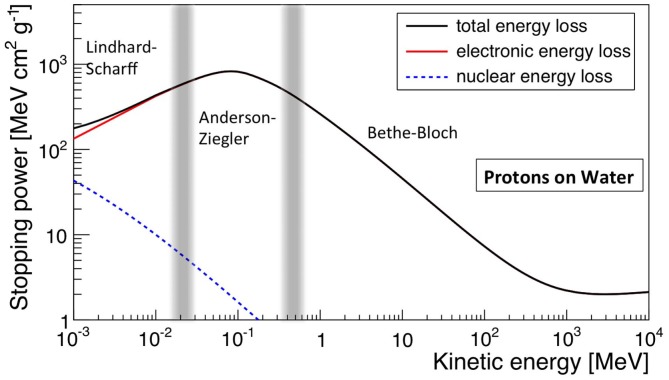
**Stopping power (d*E*/d*x*), in MeV cm^2^ g^−1^, for protons in water as function of kinetic energy**. The total, electronic, and nuclear stopping power are shown, as well as the characteristic regions. Made using NIST data (18).

**Figure 2:**
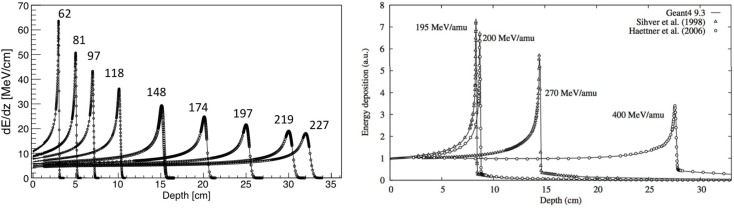
**Left:** stopping power of protons with various energies in MeV, showing CNAO data and FLUKA simulations. Adapted from Ref. (19), with permission. **Right:** stopping power of carbon ions with various energies including data and Geant4 simulations. Reproduced from Ref. (20), with permission.

The electronic stopping power as function of the kinetic energy of protons impinging on a water target is shown in Figure 1, where the various regions mentioned above are indicated. Also indicated is the nuclear stopping power resulting from Coulomb interactions of the incident particles with the atomic nuclei, which is seen to contribute very little to the total stopping power. In Figure 2, the energy loss as function of depth is given for protons (left) and ^12^C ions (right) for various energies. The growing energy loss with decreasing particle velocity described by the Bethe-Bloch formula causes the characteristic Bragg peak.

The range *R* of a particle beam is the depth in the medium, at which half of the particles undergoing electromagnetic interactions have stopped. In practice, a dose measurement is used, where the range is defined as the distal 80% point of the Bragg peak (8).

The Bragg-peak is never perfectly sharp. First of all, the ionization energy loss of a charged particle traversing a medium is a stochastic process, so that the actual range of each single particle deviates from the expected mean value. This longitudinal widening of the Bragg peak is known as *range straggling*. Second, the beam is never perfectly mono-energetic. Depending on the machine, the spread is of the order of 1% of the energy (5).

Continuous ionization energy losses of charge particles are typically modeled in Monte Carlo codes analytically down to about 2 MeV, based on a continuous-slow-down-approach (CSDA) building on the Bethe-Bloch equation, but including relevant correction factors in *Z*_p_. Below 2 MeV parameterizations are usually used. Energy straggling is partly taken into account by the emission of delta-rays, and it can be modeled using a statistical approach to include fluctuations, for instance, based on Gaussian fluctuations or the Landau or Vavilov theories (13).

#### 2.1.2 Multiple Coulomb Scattering

**Figure 3:**
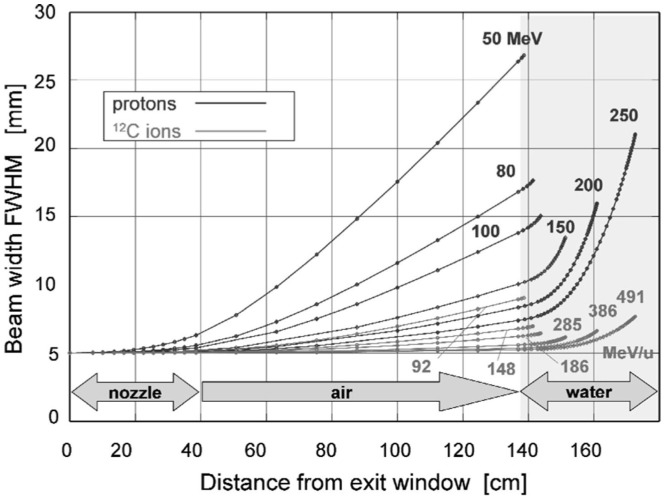
**Lateral scattering (FWHM – full width at half maximum) as function of distance from the beam exit window for various proton and carbon energies, calculated for a nozzle based on the GSI therapy facility**. The beam enters the patient body at a distance of 1.40 m from the exit. Reproduced from Ref. (25), with permission.

Besides inelastic collisions with the atomic electrons, a charged particle also suffers numerous elastic Coulomb scatterings from the nuclei themselves. The energy loss as a result of multiple Coulomb scattering (MCS) is negligible, but it is nevertheless important for dosimetry, because it causes lateral broadening of the pencil beam. Theoretical calculations of the scattering angle are highly complex. One of the most complete derivations was performed by Molière (21), and various calculations in order to derive more practical formulas were performed afterwards, for instance by Lewis (22), Highland (23), and Gottschalk (24). Due to the Central Limit Theorem, the probability distribution of the net angle of deflection of a particle in a thick material is very nearly Gaussian, resulting from the sum of many small random deflections. An approximation for the probability distribution for the net angle of deflection by MCS in a material was derived by Highland (23), and can be approximated by a Gaussian distribution with a width given by:

(7) $$\theta_{0}=\frac{14.1\text{MeV}}{\beta \mathrm{cp}}Z_{\text{p}}\sqrt{L/L_{0}}\left[1+0.038\text{ln}\left(L/L_{0}\right)\right]$$

where *L* the thickness of the scattering material and *L*_0_ the radiation length. The gaussian description is not perfect, and the presence of large-angle tails, which are the result of single scatters in the target, are not quite negligible and are typically simulated in MC codes. Also, for heavy particles, nuclear form factors should be applied, as well as Fano corrections (13). Figure 3 shows the lateral beam widening for proton and carbon projectiles.

Multiple Coulomb scattering is generally modeled in Monte Carlo codes through a combination of “condensed” MC simulations methods (most frequently based on Molière or Lewis theory, the latter also allowing to predict moments of lateral displacement) and the possibility for single large-angle scatterings. While in the former method, only the net displacement, energy loss, and change of direction at the end of the particle track are calculated, the latter allows simulating discrete single scatterings.

### 2.2 Nuclear interactions

Charged particles can also suffer nuclear interactions with the material nuclei. These interactions contribute significantly less to energy losses than electromagnetic processes. Still, they are highly relevant for range verification methods, as we will see below. Contrary to electromagnetic interactions, no rigorous models exist to describe them. In the following, we briefly describe the common approaches to model nuclear interactions, as adopted by most state-of-the-art MC codes.

#### 2.2.1 General Aspects

In most MC codes, nuclear interactions are handled in two separate steps. First, the probability that a nuclear event happens is sampled, based on nuclear cross sections. Depending on the incident particle and energy, these can be calculated “on-the-fly,” i.e., on an event-by-event basis using for instance parameterized formulas and/or physics models, or by “looking-up” a pre-evaluated cross section from a nuclear database. Examples of large nuclear databases are the Evaluated Nuclear Data File (ENDF) (26), the Japanese Evaluated Nuclear Data Libraries (JENDL) (27), and the Exchange Format (EXFOR) database (28). These contain data of thousands of experiments stored in a given format, which can be accessed from all over the world.

Once an event happens, the outcome must be sampled. This can be done with appropriate nuclear interaction models, or by using information on spectra and angular distributions from evaluated nuclear databases. As we will see in Section 3, different transport codes use data libraries in different energy regions, and for reactions induced by different projectiles.

Nuclear interactions (collisions) can be divided into:

- Elastic collisions: here kinetic energy is conserved, and the nucleus stays intact. This is similar to multiple Coulomb scattering, but now due to strong rather than electromagnetic interactions. Such interactions are not occurring so frequently, but still they cause a certain amount of broadening of the beam, which is simulated with Monte Carlo codes.
- Inelastic collisions: here, a more violent reaction between projectile and target occurs, where total kinetic energy is not conserved. The projectile may knock out secondary particles (protons, neutrons, deuterons, *α*’s, etc.) from the nucleus and break into fragments if it is an ion.

The probability *P*(*x*) of *not* having undergone a given nuclear interaction after traveling distance *x* in a material is given by:

(8) $$P\left(x\right)=\frac{N\left(x\right)}{N\left(0\right)}=e^{-\frac{x}{\lambda_{\text{int}}}},$$

where *N*(0) is the number of incident particles, *N*(*x*) the number of incident particles after a distance *x*, *λ*_int_ the mean free path or interaction length. The latter is given by $\lambda_{\text{int}}=\frac{A_{\text{t}}}{N_{\text{A}}\sigma \rho }$, where *σ* is the total cross section. Since there are some important differences in modeling the nuclear interactions for proton and heavier ions, we discuss them separately.

#### 2.2.2 Nuclear Interactions of Protons

**Figure 4:**
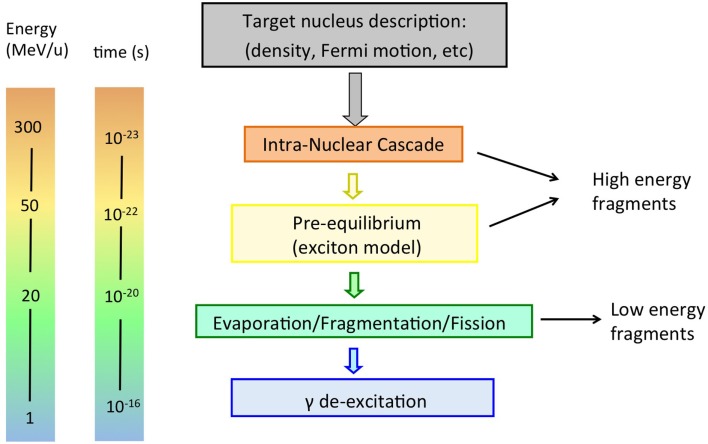
**Schematic display of stages in a nucleon-nucleus interaction relevant for radiotherapy, together with time scale and energy of interacting particle**.

**Figure 5:**
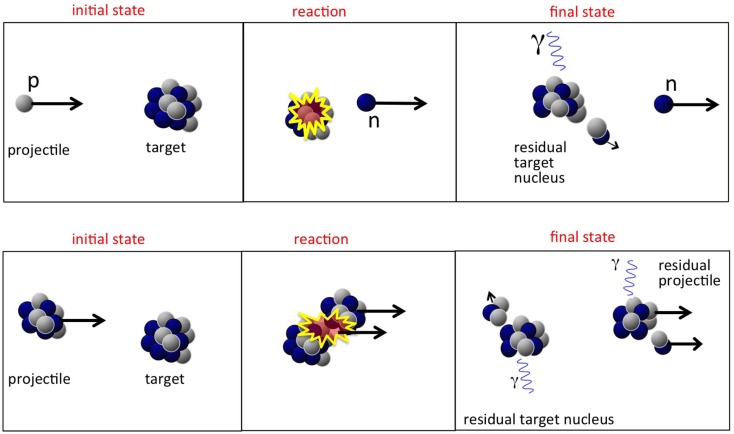
**Top:** sketch of a possible nucleon-nucleus reaction in proton therapy, whereby a neutron is created. **Bottom:** sketch of nucleus-nucleus reaction in heavy ion therapy, with creation of light fragments.

It is usually assumed that a proton hitting the atomic nucleus initiates a series of nucleon-nucleon collisions, which leads to emission of protons, neutrons, light fragments, and to equilibration of the remnant nucleus. This process can be described as a sequence of three stages (29, 30), displayed schematically in Figures 4 and 5 (top):

- (Generalized) Intra-nuclear cascade (INC)^1^: this model is commonly used to describe nuclear interactions of nucleons with energies above 50 MeV to hundreds of GeV. Originally proposed in the fourties by Serber and Heisenberg (31), and successfully implemented in the sixties by Bertini et al. (32), it forms the basis for nuclear interactions in most modern MC codes. The basic idea is that the incident particle interacts with quasi-free nucleons in the target nucleus through a series of two-body interactions. The target nucleus is modeled as a Fermi gas of cold, free, nucleons. The nucleons inside this intranuclear medium are accounted for by a nuclear density distribution, a nuclear potential, and the Pauli exclusion principle. This “free” nucleon approach is justified if the De Broglie wavelength *λ*_h_ of the incident particle is much smaller than the average distance <*d*> between the nucleons in the material nucleus, and much smaller than the mean free path *λ*_N_ inside the nucleus:
  (9) $$\lambda_{\text{h}}=\frac{2\pi \hbar }{p}\ll \langle d\rangle ={\left(\frac{3}{4\pi \rho_{\text{N}}}\right)}^{1/3}$$
  (10) $$\lambda_{\text{h}}=\frac{2\pi \hbar }{p}\ll \lambda_{\text{N}}=\frac{1}{\sigma \rho_{\text{N}}}$$
  where *σ* is the proton-nucleon cross section and *ρ*_N_ is the intranuclear density (typically 0.17 nucleons/fm^3^ at the center of nuclei). Another requirement for this approach to be valid is that the time in which a collision happens is smaller than the time between the collisions, so that they take place independently. For radiotherapeutic energy ranges, it is not immediately obvious that this approach is valid. For instance, a proton of kinetic energy 250 MeV has *λ*_h_ ~ 1 fm, which is roughly the same as <*d* >, making the condition in Eq. 9 invalid. It turns out that the INC model works surprisingly well at much lower energies than one would expect, thanks to quantum effects that increase the effective mean free path of nucleons in the nuclear medium, like Pauli blocking, nucleon-nucleon correlations, etc.

Once a nuclear interaction happens, the code has to model the outcome. For therapeutic proton energies, only elastic scatterings occur because these energies below the pion production threshold of 290 MeV. The final state particles in the scattering process are called secondaries. The time in which they are produced corresponds to the time-scale of strong interactions: 10^−22^−10^−23^ s. The secondaries have high energy and can scatter again in the same nucleus, or escape, etc. Not only protons and neutrons can be emitted, but also light nuclear fragments of high energy, through the *coalescence* mechanism, in which emitted nucleons, which are near in phase space, are grouped. All particles are tracked down until they are all below a given energy threshold, usually a few tens of MeV. This process is called an *intranuclear cascade*. The description of this process is highly complex, because all secondaries must be transported through the nuclear medium correctly, requiring accurate descriptions of the nuclear density, quantum effects, the nuclear potential, binding energy, Fermi-motion, and so on. A thorough description of the physics and useful references can be found in Ref. (29, 30).

- Pre-equilibrium: in this stage, the energy of the particles in the cascade has reached a lower limit, usually a few tens of MeV, but the nucleus is not yet in thermal equilibrium. It is commonly modeled in MC codes according to the exciton model (33, 34), a semiclassical model introduced to explain high-energy emitted particles in nuclear reactions. The evolution of the nuclear reaction is also pictured as successive nucleon-nucleon collisions, but within a particle-hole, or “exciton,” formalism, where nucleons are excited from within the Fermi sea, leaving a hole. Protons, neutrons, and light fragments (through coalescence) are emitted and the residual nucleus is left in an equilibrium state, with a certain excitation energy shared among the remaining nucleons.
- De-excitation step: depending on the mass of the target nucleus and on the energy left, the nucleus can dissipate its remaining energy in several ways.
  - Nuclear evaporation according to the Weisskopf-Ewing approach (35). Here, light fragments (*α*, *d*^3^, H^3^, He) with kinetic energies of a few MeV can be successively emitted from the excited nucleus, similar to evaporation from a hot system.
  - Fission: the excited nucleus breaks into two fragments: applies to high *Z* nuclei only, roughly Z ≳65. Apart from implants, high-*Z* nuclei are not found in the human body and this process is hardly relevant here.
  - Fermi-breakup (36): this mechanism applies to light nuclei (usually A ≲16), where the excitation energy of the excited nucleus may be larger than the binding energy of some fragmentation channels. In this case, the excited nucleus disassembles in one step into smaller fragments. Fermi-breakup is relevant for radiotherapy, because the human body is mainly composed of low-*Z* nuclei.
  - Gamma emission: What’s left after the previous stages is a residual nucleus, with may be still somewhat excited. The final excitation energy is given off through the emission of *γ* rays.

The first two steps are often referred to as “dynamic” stages of the process, with an overall time scale of about 10^−22^ s, while the last step is “slow,” typically 10^−18^−10^−16^ s. It must be noted that the emission of secondary particles in proton therapy is entirely due to the target nuclei, as was displayed in Figure 5 (top).

#### 2.2.3 Nuclear Interactions of Heavy Ions

The fundamental difference between nucleus-nucleus reactions and nucleon-nucleus reactions is that the incoming nucleons are not free. This has some important phenomenological implications. Most models for nucleus-nucleus interactions are variants of the “abrasion-ablation” model. During the fast stage (abrasion, time scale ~10^−22^−10^−23^ s), the projectile and target nuclei overlap, resulting in a kind of reaction zone. An excited quasi-projectile is formed with much of the initial velocity, a quasi-target fragment at rest, and several excited light fragments. During the slow step (ablasian, time scale ~10^−18^−10^−16^ s), the remaining projectile, target and light fragments de-excite by evaporating light nuclei or fragments. It must be noted that in this case *both* target and projectile-nuclei can fragment, as opposed to proton irradiation, where only the target-like nuclei can fragment. This is illustrated in Figure 5, showing a sketch of a nucleus-nucleus interaction. The projectile fragments travel further in the forward direction, loosing energy through ionization and undergoing further interactions. These fragments have approximately the same velocities and directions as their mothers, but larger ranges than the primary ions because range scales with *A/Z*^2^. This leads to the characteristic tail beyond the Bragg peak (see Figure 2, right). The evaporation products from the projectile fragments are evaporated isotropically in the reference frame of the projectile fragment. The target fragments have short ranges and high stopping power, and their evaporation products are evaporated isotropically in the reference frame of the target fragments.

For describing the dynamic stage of the reaction, various models have been developed, differing mainly in the treatment of the nuclear field affecting the propagation of the particles inside the nucleus.

- Intra-nuclear cascade model, for high-energy nuclei, with energies above about 100 MeV/u. The description is similar to what was outlined above for protons. The highly excited nuclei loose energy through a series of two-body reactions and scattering off quasi-free nucleons. More than one nucleon-nucleus interaction can take place in one nucleus-nucleus collision.
- Quantum molecular dynamics (QMD), for energies from 50 to about 400 MeV/u. This model can be seen as sophisticated form of the INC model. Here, each nucleon is described by a gaussian wave packet, and all nucleons in the projectile and target nuclei are participants in the collision process. By minimizing the Hamiltonian that describes nucleon-nucleon-interactions in the overlapping projectile and target nuclei, it predicts the formation of heavy or light nuclei and secondary protons and neutrons. Because of their complexity, these models are generally much slower in MC codes than the normal INC model.
- Boltzmann-Master-Equation (BME): this is a sophisticated model to simulate the pre-equilibrium stage, describing the thermalization of composite nuclei for projectiles with energies below 100 MeV/u down to the evaporation/fission/breakup stage. Based on a set of time-dependent transport equations, BME describes how a statistical state far from equilibrium evolves to an equilibrium state, through a sequence of two-body interactions and emission of unbound particles (neutrons/protons) and clusters (heavy/light nuclei).

For the de-excitation phase in nucleus-nucleus interactions, the same models as those already described for nucleon-nucleus interactions are used: evaporation, fission, Fermi-breakup, and gamma emission.

#### 2.2.4 Consequences of Nuclear Reactions

There are some important practical consequences of nuclear interactions in hadron therapy:

- Nuclear reactions cause a significant loss of beam fluence. The number of particles left at depth *x* depends on the inelastic nuclear cross section through the relation in Eq. 8. For instance, for 160 MeV protons hitting a water target, roughly 20% of the incident protons will be lost at the Bragg peak position at about 16 cm (8). For carbon ions, this number is much higher: for 290 MeV/u carbon ions impinging on water, in average 50% of the ions have undergone a nuclear reaction at the end of range at around 16 cm (37).
- The dose distributions are modified. In the buildup region of the Bragg curve, secondary particles contribute considerably to the total energy deposition. Moreover, the height of the Bragg peak is modified. In addition, in case of heavy ion irradiation, dose is also delivered beyond the Bragg peak. Finally, the low energetic secondary particles (including neutrons), which are typically emitted at larger angles, contribute to the “low dose envelope” around the beam. Even though the dose contribution of a single pencil beam is tiny, when combining thousands of them, the dosimetrical and biological effects cannot be neglected (38, 39), because the charged fragments can be particularly damaging (see Section 2.3).
- Various types of secondary particles are produced, which can be used for particle range verification. All three stages of the nuclear reactions contribute to the production of secondaries. The INC and pre-equilibrium stage mostly lead to production of high-energy secondary particles (with energies that may exceed even a hundred MeV), emitted mostly in the forward direction in the laboratory frame. The slow part of the nuclear interaction leads to lower energy secondaries, emitted more or less isotropically in the center-of-mass frame of the mother nucleus. In the case of ion projectile emissions, this implies mostly forward in the laboratory system due to the kinematic boost. Three types of secondaries are used for range monitoring in hadron therapy:
  - *β*^+^ emitting isotopes: among the many fragments produced during irradiation are *β*^+^ emitting fragments. Table 1 shows the most frequently produced *β*^+^ emitters in a human body treated with proton therapy together with their half-lives and the incident proton threshold energy. Other reactions are listed elsewhere (40). Depending on the value of their half-life, the *β*^+^ emitters emit at a later time a positron, which after traveling a small distance (of order mm), annihilates with an electron in the medium into two coincidence gammas of 511 keV. These can be measured with a PET detector, enabling to extract information about the beam path. In Section 4.2, we come back to the PET method.
  - Prompt gammas, which accompany the nuclear reactions along much of the proton path. Prompt photons are emitted with energies ranging from 0 to about 10 MeV. More details and energy spectra will be discussed in Section 4.3.
  - Charged fragments are produced, which can possibly be measured and used for verifying particle range, will be discussed in Section 4.4. The secondary particles are usually characterized by energy spectra and double differential cross section measurements, i.e., particle fragment yields as a function of their energy, at different angles.

**Table 1:** **Most frequently occurring nuclear reaction channels for positron emitter production in proton therapy**.

*β*^+^-emitter	Half-life (min)	Reaction channel	Threshold energy (MeV)
^15^*O*	2.037	^16^O(p,pn)^15^O	16.79
^11^*C*	20.385	^12^C(p,pn)^11^C	20.61
		^14^N(p,2p2n)^11^C	3.22
		^16^O(p,3p3n)^11^C	59.64
		^16^O(p,*α* d)^11^C	27.50
^13^*N*	9.965	^16^O(p,2p2n)^13^N	5.66
		^14^N(p,pn)^13^N	11.44
^30^*P*	2.498	^31^P(p,pn)^30^P	19.7
^38^*K*	7.636	^40^Ca(p,2p2n)^38^K	21.2

*Adapted from Ref. (40, 41)*.

### 2.3 Dosimetry considerations

The absorbed dose *D* in a patient is related to the stopping power by Gottschalk (8):

(11) $$D\left(\text{Gy}\right)=1.602\times 10^{-10}\times F\frac{\mathrm{dE}}{\mathrm{dx}}\frac{1}{\rho }$$

where *F* is the particle fluence in cm^−2^, *ρ* the target density in g/cm^3^, and d*E*/d*x* the stopping power in MeV/cm. For clinical dose calculations in particle therapy, the mass stopping power ((d*E*/d*x*)/*ρ*) is obtained from stoichiometric calibrations curves, which link CT Houndsfield units in each voxel to mass stopping power values, such as proposed by Schneider et al. (42).

To estimate biological effects, considering the physical dose proves to be inadequate because biological damage caused by radiation depends, e.g., strongly on the particle type and energy. Although it is beyond the scope of this article to discuss biological effects, a few concepts are relevant. The linear energy transfer (LET) of a particle beam is the energy deposited locally per unit path length, on microscopic level. Particles with high-LET such as ^12^C ions cause more lethal damage to the cancerous cells than proton or photon beams. Therefore, each ion type has a relative biological effectiveness (RBE) assigned, defined as the ratio of biological effectiveness of one type of ionizing radiation relative to X-rays, given the same amount of absorbed energy. The RBE in the Bragg peak region is close to 1 for protons (43) and between 3 and 4 for Carbon ions (44). For the latter, it must be included in treatment planning (45).

### 2.4 Modeling uncertainties and validation

Two major uncertainties in calculating the stopping power and particle range in MC codes are the material density and the ionization energy *I*_e_ in water. Stopping powers deduced from CT scans suffer additionally from uncertainties like the calibration of the CT scanner, conversion HU to stopping power. The dependence on *I*_e_ is only logarithmic, but variations in the evaluated value give range uncertainties of about 1–2% for mono-energetic proton beams (46), and even larger range uncertainties were found for in patient tissues (47). In addition, the accuracy of stopping power and range calculation depends also on other factors, like the accuracy of the knowledge on the particle energy of the machine, the precision and accuracy of the measurement device, the step sizes in the MC code, the accuracy of the beamline description, the treatment head, and so on.

Stopping power models in Monte Carlo codes used in medical physics are usually benchmarked with standard quality assurance (QA) in-house dosimetry measurements on homogeneous and heterogeneous targets, typically performed with ionization chambers, calorimeters, and Faraday cups. With the latter, it is possible to measure the longitudinal charge distribution of primary and secondary particles, and to separate the nuclear interaction component from the electromagnetic component. Lateral scattering models can be validated by measuring lateral dose profiles. The validation of MC codes at therapeutic energies is important, because many MC codes have originally been developed for high-energy physics, pertaining to different energy regions.

Uncertainties in modeling nuclear interactions come mostly from uncertainties in cross sections, whereby total cross sections and double differential (energy and angle) cross sections are most relevant. The size and impact of these uncertainties is strongly dependent on the purpose of the measurement: dosimetry, shielding, non-invasive range monitoring, and so on. Especially when parameterizations used in MC codes are based on a few measurements or when no data are available at all and models must be relied on, uncertainties can be substantial, as is the case for instance for production of *β*^+^ emitters. Additional uncertainties apply when tissue composition is deduced from CT scans. We come back to this in Section 4, where non-invasive range verification techniques are discussed. Uncertainties on total cross section calculations are quantified by Sihver et al. (48), presenting comparisons of various nuclear interaction models with each other and with experimental data in an energy range relevant for radiotherapy.

Although dosimetry can certainly help to validate nuclear interaction models, it is often impossible to perform direct experimental validation of the nuclear models in MC codes. A first validation of nuclear interaction models, which can be done in-house, is Faraday-cup measurements. Charged fragment production is generally validated with experimental data collected over the years of various thin and thick target measurements, including both integral and differential quantities. An example of a recent experiment contributing to the collection of relevant data is the Fragmentation of Ions Relevant for Space and Therapy (FIRST) experiment (49), aiming at cross section measurements for projectile-target combinations and energies relevant for ion beam therapy. Selected examples of the validation of MC codes relevant for non-invasive range monitoring will be given in Sections 3 and 4.

## 3 MC Codes

In this section, we summarize relevant features of the three most frequently used MC codes in hadron therapy studies: Geant4, FLUKA, and MCNP6/X. For each, we discuss transport and interactions, as well as the validation for hadron therapy simulations: depth-dose profiles, nucleon-nucleus interactions, and nucleus-nucleus interactions. While we describe in this section the general aspects like dose calculations and secondary particle production, in Section 4 we will narrow the focus to range monitoring. Extensive reviews about the general use of MC codes in radiotherapy can be found elsewhere (10, 11).

### 3.1 FLUKA

FLUKA (50, 51) (FLUktuierende KAskade) is a general purpose MC generator for the transport and interactions in matter of particles from a few keV to cosmic ray energies. Originally developed for high-energy physics, it is nowadays widely used for shielding applications, detector design, cosmic ray showers, and medical physics. The code is written in FORTRAN.

#### 3.1.1 Particle Transport and Interactions

Charged particle transport is done through a Multiple Coulomb scattering algorithm (52) based on Moliere’s theory, with Fano corrections, and supplemented by an optional single scattering method. Ionization energy losses are based on statistical approach reproducing ionization and fluctuations therein (53, 54), including *δ* ray emission and energy straggling.

Hadron-nucleus interactions are modeled in FLUKA with the PEANUT (Pre-Equilibrium Approach to NUclear Thermalization) model (30, 51), which is valid in a very broad energy range, from reaction threshold up to a few tens of TeV. This model simulates the first two stages of nuclear reactions described in Section 2.2.2. The intranuclear cascade (INC) stage includes many sophisticated features, including nuclear potential effects like curvature of the path, and quantum effects, like Pauli blocking, nucleon-nucleon correlations, etc. The pre-equilibrium stage is based on the exciton formalism from Blann (34). A coalescence algorithm is used for emission of composite projectiles. PEANUT ends when all particles are below a certain threshold, of the order of 10–20 MeV. The final relaxation step in FLUKA includes models for simulating nuclear evaporation, fission, Fermi-breakup (*A* ≤ 17), and gamma emission. Recently, a direct deuteron formation mechanism has been added in FLUKA (55). Cross sections are based on parameterized fits and tabulated data, when available. Otherwise, they are calculated with appropriate models. For low energy neutron transport, FLUKA is linked with ENDF and JENDL.

Nucleus-nucleus interactions are handled in FLUKA through interfaces to event generators, which simulate the dynamic part of the nucleus-nucleus interaction. Between 100 MeV/u and 5 GeV/u, a relativistic quantum molecular dynamics (rQMD) model is used (56). Below 100 MeV per nucleon, nucleus-nucleus collisions are treated following the BME theory (57). These models are all coupled to the internal FLUKA models for the slow phase of the interaction through evaporation/fission/breakup and gamma emission. For patient simulations, 3-D voxel geometries like CT scans or other 3-D descriptions of human body can be read by FLUKA. FLUKA Advanced InteRface (FLAIR) is a modern user-friendly interface to FLUKA, which facilitates editing input files, execution of the code, and visualization of the results.

#### 3.1.2 Validation

Depth-dose profiles are important to check the validity of both the electromagnetic and hadronic physics. FLUKA simulations have been thoroughly validated with experimental depth-dose data for protons and heavy ions (19, 51, 58). An example for protons is given in Figure 2 (left), showing the comparison of measured depth dose profile and the FLUKA simulation for various energies.

**Figure 6:**
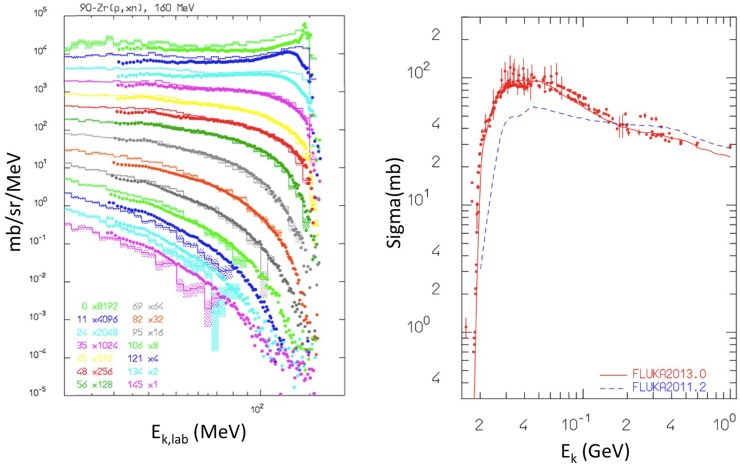
**Left:** double differential energy spectra of emitted neutrons at different angles, from 160 MeV protons impinging on a Zr target. Histograms are FLUKA results, points are experimental data from Ref. (61, 62). Reproduced from Ref. (51), with permission. **Right:** cross section for ^nat12^C(*px*)^11^C production from protons irradiation as computed with fluka2013.0 (red, upper curve), and fluka2011.2 (blue, lower curve) compared with data retrieved from the EXFOR library (28). Reproduced from Ref. (55), with permission.

Hadronic interactions in FLUKA have been extensively benchmarked against a variety of experimental data (51, 55, 59, 60). An example relevant for proton irradiation is shown in Figure 6 (left), showing the simulated and measured secondary neutron double differential energy spectra, resulting from 160 MeV protons impinging on a Zr target. Still for proton irradiation, Figure 6 (right) shows the validation of the production of the *β*^+^ emitter ^11^C from proton irradiation of a ^12^C target. Also, longitudinal charge distributions of proton beams measured with Faraday cups have been compared with FLUKA simulations to test the nuclear models (54).

**Figure 7:**
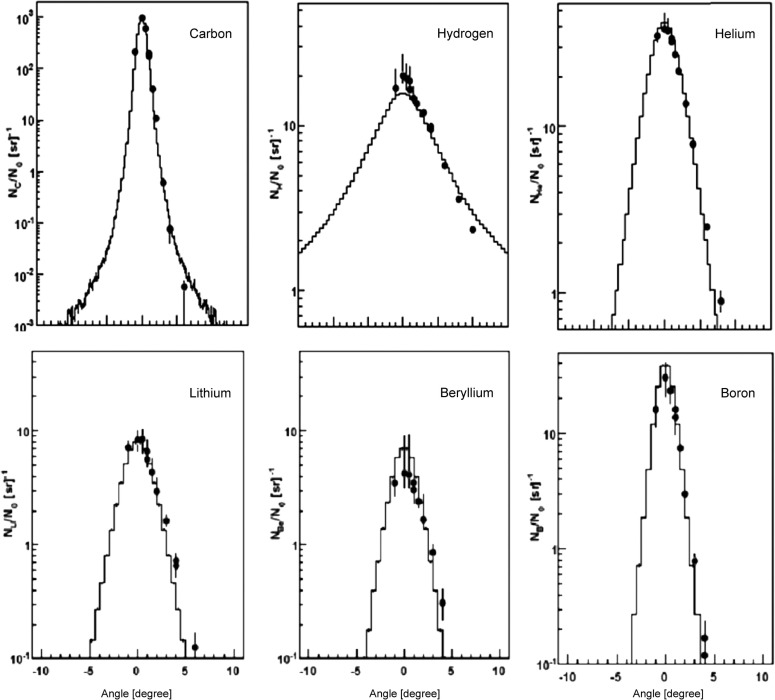
**Measurements (63) (circles) and FLUKA simulations (histograms) of angular distributions of a carbon beam (left top) and secondary fragments (others) measured behind a 15.9 cm water target, irradiated with a 400 MeV/u ^12^C ions**. Reproduced from Ref. (60), with permission.

For ^12^C irradiation, Figure 7 nicely demonstrates the reliability of the nucleus-nucleus interaction models. In this study by Mairani et al. (60), simulations were compared with measurements (63) of secondary particles behind a 15.9 cm water target, irradiated with 400 MeV/u ^12^C-ions. The transmitted primary beam and the angular distribution of the secondary fragments were measured. This plot demonstrates that the MCS model together with the nuclear interaction models describes absolute yield and angular distribution of the ^12^C beam and the produced fragments.

The performance of FLUKA to simulate the specific reaction products like *β*^+^ emitters and prompt *γ*’s will be shown in Section 4.

### 3.2 Geant4

Geant4 (64) is an open-source modern MC toolkit for simulating the passage of particles in matter, written in C++. Originally designed for the LHC experiments, its use has been extended to medical physics, space science, nuclear physics, accelerator physics, and so on. A set of standard physics settings for proton therapy was proposed by Jarlskog and Paganetti (65), but this prescription has been modified. Below we discuss the most relevant Geant4 physics models that are commonly used for hadron therapy simulations. Details and references can be found in the Geant4 manual (64) and in dedicated lectures (37).

#### 3.2.1 Particle Transport and Interactions

Electromagnetic energy losses for hadron therapy studies are usually based on the so-called “electromagnetic standard package option 3” list. Protons with energy above 2 MeV are in Geant4, simulated according to the Bethe-Bloch formula, while below 2 MeV stopping power parameterizations are used. The multiple scattering model is based on Lewis theory (22). For range straggling, appropriate fluctuation models are provided.

Concerning hadronic interactions, Geant4 offers various models. Starting with protons, the dynamic part of inelastic nuclear interactions can be simulated with the Binary Cascade Model (BIC). This model simulates the INC stage described in Section 2.2.2 and includes relevant nuclear potential effects and quantum effects, similar to FLUKA. This can be followed by a pre-equilibrium stage (“precompound” model), which is based on the exciton formalism from Griffin (33). Geant4 also offers alternative models to BIC: the intra-nuclear cascade Liège (INCL) model from Boudard et al. (66), and the Bertini-model (32), differing in many aspects, including the treatment of the nuclear potential, nuclear density, and coalescence. For simulating the de-excitation step, Geant4 includes several possibilities: standard evaporation model based on the Weisskopf-Ewing approach (for emissions of nucleons and light fragments, up to ^4^He), generalized evaporation model (GEM, including also emissions of heavier fragments), fission, multi-fragmentation (for nuclei with excitation energy above 3 MeV/u), Fermi-breakup (*A* < 17 and *Z* < 9), and gamma emission. To evaluate nuclear cross sections, Geant4 is linked to various nuclear databases, including ENDF, and when no data are available calculations are used.

For heavier projectiles like ^12^C, Geant4 provides various possibilities. The dynamic stage of the nucleus-nucleus interactions can be simulated with the G4BinaryLightIonReaction (BLI) model, a semi-classical INC model, but extended to take into account that more than one nucleon participates in the reaction. Geant4 also offers the sophisticated G4QMDReaction model, a newly implemented nucleus-nucleus interaction model based on QMD. Alternatively, the INCL++ (Intra-Nuclear-Cascade Liège) model can be used. All of them must be coupled to the aforementioned de-excitation models.

#### 3.2.2 Validation

Starting with protons, good agreements between measured and simulated depth-dose profile were reported in Ref. (67, 68). Geant4 was also shown to satisfactorily describe lateral beam widening (68), although others reported disagreements (67). Hadronic interactions were also validated against measured Faraday cup data (65). For carbon ion therapy, various groups reported good agreements of dose-depth profiles, including the fragmentation tail (20, 69–71), an example of which is shown in Figure 2.

Several authors investigated the validity of nuclear fragmentation models for particle therapy. Much work has been reported by Pshenichnov et al. (69, 72, 73), making use of a dedicated framework MC model for Heavy-Ion Therapy (MCHIT). Comparisons between simulated and measured depth-dose curves, nuclear fragmentation build-up curves, angular distributions, and yields of secondary particles (including *β*^+^ emitters) were performed for protons and heavier ion beams impinging on homogeneous targets, leading to improvements in the nuclear modeling in Geant4. MCHIT is also currently being used for validating microdosimetric models (74).

More recently, validations of the newly implemented Geant4 models relevant for nucleus-nucleus interactions have been performed. Böhlen et al. (59) reported a good agreement of the QMD model with data in describing nuclear fragmentation in carbon irradiations. Also, Robert et al. (75) studied depth-dose profiles and secondary particle production in proton and carbon therapy for Geant4 and FLUKA. Comparing depth-dose profiles and energy spectra at various angles of charged particles and prompt gammas, they identified the main differences between the codes. Absolute yields were found to differ by roughly 20 and 100% for *β*^+^ emitters and prompt photons. Also, De Napoli et al. (76) and Dudouet et al. (77) presented comparisons between measured and simulated double differential energy spectra, including the BIC, QMD, and INCL++ models, for mono-energetic carbon beams impinging on various thin targets. None of the models could satisfactorily describe yields, angular and double differential energy distributions.

Geant4 validation studies for *β*^+^ and prompt gamma emissions will be discussed in Section 4.

#### 3.2.3 Geant4-Based Applications

Because the high level of experience required to use Geant4 has proven to be a barrier for clinical usage, several user-friendly tools making use of the Geant4 physics have been developed.

Geant4 Application for Emission Tomography (GATE) (78) is an open-source MC framework making use of the Geant4 libraries. Originally dedicated to PET and SPECT systems, GATE also offers the possibility for hadron therapy simulations, including *in vivo* range monitoring using PET (79). GATE allows simulating very complex geometries like commercial PET or SPECT scanners, time dependent quantities phenomena, and it also offers image reconstruction tools.

Another example is Tool for Particle Simulation (TOPAS) (80), a simulation tool dedicated to proton therapy simulations. Recently, an extensive validation of TOPAS has been performed for proton therapy treatments with the passive scattering technique at MGH (81), based on routinely performed quality assurance (QA) measurements (lateral and longitudinal dose measurements, and so on). TOPAS has been used for range verification studies with prompt gamma imaging, as will be described in Section 4.3.2.

Particle therapy simulation framework (PTSIM) (82) is a Geant4 software tool which can be used to model a complete hadron therapy treatment, including beam delivery system, a treatment head, and patient data obtained from CT images. It has been used for carbon therapy simulations with the facilities in Japan.

Finally, Geant4-based architecture for medicine-oriented simulations (GAMOS) (83) is another Geant4-based simulation framework aimed at nuclear medicine simulations, including hadron therapy applications.

### 3.3 MCNPX/6

#### 3.3.1 Particle Transport and Interactions

Monte Carlo N-Particle version 6 (MCNP6) (84) is a general purpose MC generator for simulating radiation transport and interactions in matter. MCNP6 is the result of merging and extending the older MCNP5 (85) and Monte Carlo N-Particle eXtended (MCNPX) (86) codes, written in FORTRAN.

Continuous ionization energy losses are modeled analytically according to the Bethe-Bloch formula, using ionization potentials recommended by the ICRU data. Energy straggling is based on the Vavilov straggling model (87), and multiple scattering is based on Rossi’s theory (88).

At present, MCNP6 has 5 different models for simulating nuclear interactions for medical physics (84): CEM03.03, Bertini, INCL+ ABLA, LAQGSM03.03, and ISABEL. For proton therapy simulations, the Cascade-Exciton Model (CEM) is currently recommended and is the default option. This model, originally proposed over 30 years ago in Dubna (89) and refined over the years, incorporates all three stages of nuclear reactions described in Section 2.2.2. The INC description includes many important aspects such as quantum effects, nuclear binding energies, coalescence, and so on. The pre-equilibrium stage is modeled with the exciton formalism, and evaporation/Fermi-breakup/fission can be used for the final relaxation step. The second model, Bertini (32), was successfully used in the past for proton therapy simulations (90–92), but performs worse in describing angular distributions of secondary particles, and is currently not maintained anymore. The third model, Intra nuclear-cascade Liége (INCL) model (66), can alternatively be used in combination with the ABLA evaporation model, but is slower.

To simulate nucleus-nucleus interactions in heavy ion therapy simulations like ^12^C, the fourth model, Los Alamos version of the Quark Gluon String Model (LAQGSM) (29) is suggested. As the CEM model, LAQGSM describes all three stages of nuclear interactions, and is valid over a large energy range even up to 1 TeV. However, the description of INC stage is entirely different from that in the CEM model, taking into account the time of interactions, the so-called “trawling effect,” etc. (see more details and further references in (29)). LAQGSM models the interactions of fast cascade particles (called “participants”) with nucleon spectators of both the target and projectile nuclei and includes also interactions between two participants. The modeling of the pre-equilibrium stage and final relaxation stage is similar to the CEM model. Finally, the fifth nuclear interaction model, ISABEL, was used in the past for simulating nucleus-nucleus interactions, but is no longer updated.

Below 150 MeV, MCNP6 uses nuclear data libraries (26, 93) evaluated from measured cross section data and calculations with appropriate nuclear models. At higher incident energies, nuclear reaction models mentioned above are used.

Concerning the usage of 3-D patient descriptions, MCNP6 includes the possibility to import 3-D voxel geometries like CT scans.

#### 3.3.2 Validation

**Figure 8:**
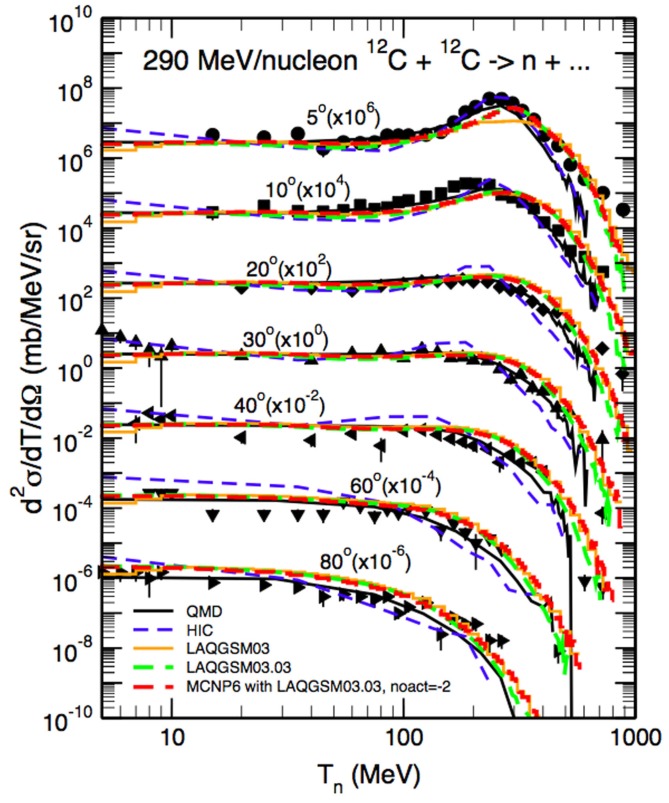
**Experimental neutron spectra (97) at different angles from a thin 12C target bombarded with a 290 MeV/u 12C beam compared with Japanese QMD calculations (98), the Bertini INC model (32) coupled to an evaporation model (HIC), the Los Alamos version of the Quark-Gluon String Model used as a stand alone code LAQGSM03.03 [see Ref. (29)], and with MCNP6 using the LAQGSM03.03 event-generator [see Ref. (94)]**. Reproduced from Ref. (94).

Longitudinal and lateral dose distributions in MCNPX and MCNP5 have been validated for proton therapy by various research groups (90–92). The modeling of nuclear interactions with MCNP6 with the CEM and the LAQGSM models has been recently extensively validated by Mashnik et al. (94–96). Fragmentation measurements from a vast set of recent and older experiments were compared to MCNP6 simulations, as documented in comprehensive Validation and Verification (V&V) Los Alamos reports (94, 95). Comparisons included total cross sections and double differential energy spectra for neutrons, protons, and light fragments (up to ^4^He) produced during irradiation of protons, light and heavy ions impinging on many different homogeneous targets. Figure 8 demonstrates an example of the validation, showing a measured double differential neutron spectrum for a thin ^12^C target bombarded with a 290 MeV/u ^12^C beam, together with MCNP6 predictions with the LAQGDM model. A very good agreement was obtained.

Relevant for proton therapy simulations is a recent validation of the CEM model in proton-induced fragmentation reactions on low-Z targets (96), focusing on intermediate proton energies (10 MeV < 1 GeV). For various fragment types produced during nuclear reactions in different targets, measurements of total inelastic cross sections, yields, excitation functions, and double differential spectra of products were compared with simulations. Overall, very satisfying agreements between data and MCNP6 were obtained.

### 3.4 Other MC codes

Here, we will only briefly report on other MC codes that are used for particle therapy.

Particle and Heavy Ion Transport code System (PHITS) (99) is a general purpose MC particle and heavy ion transport code written in FORTRAN, which can be used for simulating proton and heavy ion treatments. Ionization processes are simulated with the continuous slow down approximation. For low energy neutron induced reactions, PHITS employs the cross sections from the JENDL nuclear data library. For nuclear reactions of higher energy neutrons and other particles, various sophisticated models are available, including the Microscopic Transport Model (JAM), the JAERI Quantum Molecular Dynamics Model (JQMD), the INCL model, and the INCL-ELF model. For details and references about these models and their validation, see Ref. (99). PHITS can also determine profiles of all secondary particles, including prompt *γ*’s, and perform microdosimetric calculations.

HIBRAC is a one-dimensional simulation tool developed by Sihver and Mancusi (100) in FORTRAN, used in various clinics worldwide in treatment planning for ion beam therapy. The code is based on semi-empirical total and fragmentation reaction cross section formulas for proton-nucleus and nucleus-nucleus reactions, and models are used for calculating stopping power and energy straggling. The code can accurately predict fluence, dose, dose-average LET, track-average LET, and energy distributions as a function of the penetration depth of light ion beams in any solid or fluid target material. Predictions of the code have been validated with experimental data (depth-dose profiles, fluence) from the GSI and Chiba facilities. HIBRAC can also be used for predicting PET profiles (101), albeit only in 1-D.

SHIELD-HIT (102) is another MC code dedicated to ion therapy. It is a FORTRAN written code that is derived from the SHIELD code, originally developed at the Joint Institute for Nuclear Research in Dubna, Russia. It is possible to transport nuclei, nucleons, anti-nucleons, pions, and kaons up to 1 TeV/u and down to 1 MeV/u. It includes all processes relevant for electromagnetic interactions (straggling, MCS, ionization losses) and nuclear interactions. Nuclear fragmentation is handled by the many stage dynamical model (MSDM), simulating all three stages in nuclear reactions. The SHIELD-HIT code is primarily used in particle therapy for calculation of stopping power ratios, fluence correction factors, and anti-proton calculations.

Another MC code developed for treatment planning is the Voxel Monte Carlo for proton therapy (VMCpro) code (103), a fast MC framework, also written in FORTRAN. VMCpro simulates proton transport in human tissue based on a condensed history technique. The code is based on various approaches and parameterizations, for instance a simplified multiple coulomb scattering algorithm and density scaling functions instead of actual material compositions. Nuclear interactions are treated as corrections to electromagnetic processes. Valid results for depth-dose predictions were obtained with VMCpro, and being order of magnitude faster than for instance FLUKA and Geant, the code is a valuable tool for treatment planning.

PENELOPE is a MC code written in FORTRAN that was originally limited to the transport and interactions of photons, electrons, and positrons. It has recently been extended to protons (104) (PENH). The main motivation for the extension is to provide the medical physics community with a fast and reliable MC code for instance to perform dose calculations from treatment plans. Dose distributions obtained with PENH have been benchmarked with Geant4 (GATE) and FLUKA predictions (104).

## 4 MC Signal Modeling for *In vivo* Range Verification

### 4.1 Introduction

In this section, we discuss the three most widely researched modalities for *in vivo* non-invasive hadron therapy verification, which exploit secondary particles produced in nuclear reactions: PET (Section 4.2), prompt gamma (Section 4.3), and charged particle imaging (Section 4.4). For each of them, we introduce the technique and briefly discuss different detector types. The latter is relevant here, because it can affect the way the MC predictions are made. Furthermore, we describe examples of the MC predictions and validation procedures adopted by various research groups, and touch briefly on clinical challenges related to MC simulations. Because the focus of our review is on the physics and MC modeling, we do not discuss logistical, technical, and economical issues related to clinical integration, image reconstruction, signal analysis, clinical interpretation of detected range deviations, nor do we discuss the expected sensitivity of the techniques. These are discussed in other works (4, 10, 11). A brief comparison of the three techniques will be presented in Section 5.

Finally, other imaging methods that are currently investigated for treatment verification include proton radiography (105), proton tomography (106), and ionoacoustic imaging (107); however, we do not consider them here. The same applies for positron emitting probing beams, such as for instance investigated at the Chiba facility (108).

### 4.2 PET-based treatment verification

#### 4.2.1 Treatment Monitoring Strategies

**Figure 9:**
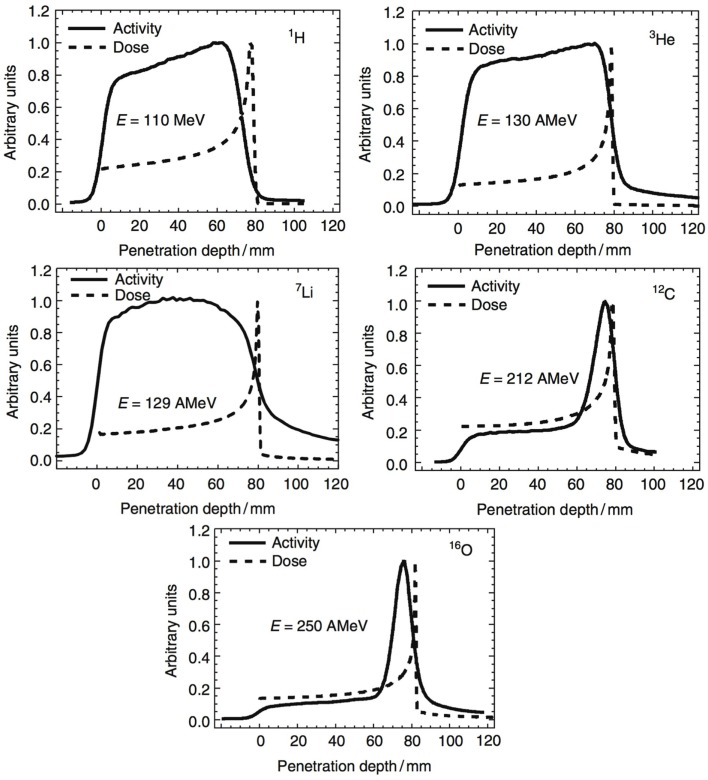
**Measured positron emission activity together with the dose distribution for irradiation of a PMMA target with different projectiles: protons, ^3^He, ^7^Li, ^12^C, and ^16^O**. Reproduced from Ref. (127), with permission.

In Section 2, we have seen how nuclear reactions of incident protons and nuclei give rise to the production of *β*^+^ emitting fragments. By detecting the two 511 keV photons by positron annihilation, spatial distributions of the *β*^+^ decay points can be obtained. Often one-dimensional profiles along the beam-axis are chosen to display the activity along the beam path. In Figure 9, such profiles are displayed for various incident beam types impinging on a PMMA target. Normalization is arbitrary here. Two things can be noticed. First, the shape of the *β*^+^ activity profiles of light beams is remarkably different from those of heavier nuclei. While for the p, ^3^He, ^7^Li ion beams, the induced activity is only due to positron-emitting *target residuals* produced all along the beam path; for the ^12^C and ^16^O beams, there is an additional contribution in the activity from *β*^+^ emitting *projectile residuals* when they stop, near the end of range, explaining the activity peak. Second, we see that no direct correlation exists between *β*^+^-activity and the dose, which is not surprising, being based on different physics processes. Nevertheless, by comparing the measured PET data with reference distributions, it is possible to estimate whether the dose was delivered successfully. Large discrepancies between expected and measured PET data indicate problems in dose delivery. Such reference distributions are generally made with MC simulations on the basis of the treatment plan, time-course of irradiation, the patient CT, detector geometry, and imaging procedure (109). The application of PET to hadron therapy dose monitoring has been studied for about 20 years and is currently a well-established, although not widely used, method. Recent reviews can be found for instance in Ref. (4, 41).

PET data acquisition strategies are usually categorized as follows:

- In-beam data acquisition, where the PET system is integrated in the beam delivery system and data are acquired during or immediately after irradiation inside the treatment room (109–117). Data-taking during irradiation is attractive, because this allows detecting activity from ^15^O and other short-lived isotopes, and avoids having to deal with biological signal washout in the human body. Moreover, no repositioning of the patient is necessary. On the other hand, disadvantages include the high costs for integration into the treatment site, geometrical limitations on the detector, and the associated image artifacts. A special challenge is acquisition during beam extraction, where background from random coincidences tends to paralyze the PET detectors (118). The most commonly chosen solution in synchrotron facilities is to take data during spill-pauses, although at cyclotrons data-taking during beam extraction was shown to be feasible (115, 119).
- In-room data acquisition, where data are acquired quickly after irradiation with a full-ring PET detector installed in the treatment room. This methodology was explored at MGH (120, 121). The advantage is that signal washout is reduced, and problems related to patient transport and repositioning are minimized. Disadvantages include a longer treatment room occupation time and difficulties in co-registration of the PET image with the planning CT.
- Offline data acquisition, where data are acquired with a full-ring PET after patient irradiation outside the treatment room (122–125). The advantages are the low costs and the complete angular coverage. However, the delay between particle delivery and monitoring greatly limits the offline method. Signal decay and biological washout processes rapidly cause signal degradation, which is difficult to model accurately (124, 126).

#### 4.2.2 PET Systems for Treatment Verification

Depending on the data acquisition strategy, different detectors can be used for PET-based treatment monitoring.

- Planar PET systems, for in-beam monitoring. A planar configuration was used at GSI for in-beam PET monitoring of patients treated with ^12^C ions (109, 111), using data acquired during spill-pauses and after treatment. Planar configurations have also been developed in Japan at Chiba (128) and have been used to monitor patient treatments with ^12^C at the Kaswhiwa facility (113). Moreover, a compact planar PET system enabling data acquisition during and after treatment has been developed at INFN and was tested on phantoms at CATANA (115, 119) and CNAO (116). Adding TOF information could help in reducing background to signal noise (129).
- Full-ring commercial PET scanners, which can be used only for monitoring after treatment. Such scanners have been used for in-room (120, 121) and offline data acquisition (122–125).
- Alternative geometries: ring-shaped PET systems are being investigated in Japan for in-beam data acquisition, including a dual-ring (130) and a full-ring (114) PET system, cut at a slant angle. The latter geometry is promising, because it would represent the first in-beam system with complete angular acceptance. Also, C-shaped and partial ring PET systems were studied with GATE for in-beam PET (131).

#### 4.2.3 Prediction of *β*^+^ Activity

Many different approaches have been used in research and clinical studies for predicting the PET activity signal. We describe them for proton, carbon, and heavier ion therapy.

Starting with protons, pioneering studies performed by Parodi et al. (122, 132) for offline PET monitoring of proton treatments at MGH were based on FLUKA simulations. Rather than relying on the internal FLUKA nuclear cross sections, the activity was calculated by folding the proton track length with external experimental cross section data (132). For activity predictions in patients, correction factors for biological washout were applied *a posteriori* on the basis of the CT scan, where regions with low, intermediate, and high perfusion were identified. The reliability of the MC predictions turned out to depend on treatment site, mostly because of problems in modeling biological washout (124).

More recent studies focusing on in-room proton therapy at MGH used Geant4 for predicting PET activity distributions (120, 121, 133). First, they compared PET measurements on homogeneous targets with MC activity predictions using different cross section data libraries. The cross section values that best described the measurements were chosen for the patient MC simulations (133). Including this tuning, the Geant4 predictions were successfully used for patient monitoring (120, 121).

A similar procedure was studied by Bauer et al. (134) for offline PET data acquisition in proton therapy at HIT. FLUKA was used to investigate the effect of directly including in-house activity measurements for homogeneous materials into the simulation. Fine-tuned cross sections turned out to reduce uncertainties, improving the modeling of proton-induced positron-emitter production.

**Figure 10:**
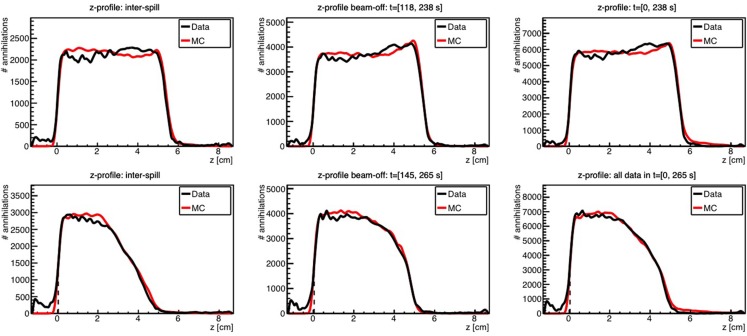
**Top:** 1-D profile along the *z*-axis of the measured (black) and MC simulated activity (red) induced by a 95 MeV proton beam impinging on a PMMA target during 118 s. **Bottom:** the same but for the 2 Gy treatment plan, delivered in 145 s. Inter-spill (left), beam-off (center), and all together (right) are displayed separately. Adapted from Ref. (116).

Kraan et al. used FLUKA to predict the PET activity measured in homogeneous targets during and after proton irradiation with an in-beam PET system at the CATANA cyclotron (119) and at the CNAO treatment facility (116). Figure 10 shows an example of measurements performed at CNAO for irradiation of a PMMA phantom with a homogeneous proton beam (top figures) and a SOBP (bottom figures), together with the FLUKA simulation, for various acquisition time intervals. A good agreement between data and MC simulations was found.

In carbon irradiation, the signal modeling is somewhat different, and other approaches have been applied. For the early PET studies on patients treated with carbon ions at the GSI facility (109, 111), a dedicated MC simulation tool (POSGEN) was developed by Pönisch et al. (135) for calculating the activity. A simplified and fast simulation approach was applied, based on the assumption that the dominant contribution to the *β*^+^ activity profile comes from projectile residuals. The calculation was split in two steps: a one-time step to calculate the activity from target residuals assuming a homogeneous medium, and a patient and fraction specific step to calculate the projectile contribution. The code used relied on cross section models developed by Sihver et al. (100) to handle nuclear interaction processes. It was validated and applied clinically for the in-beam monitoring project at GSI (110, 111, 136), and also used for modeling the PET activity for moving targets (137).

**Figure 11:**
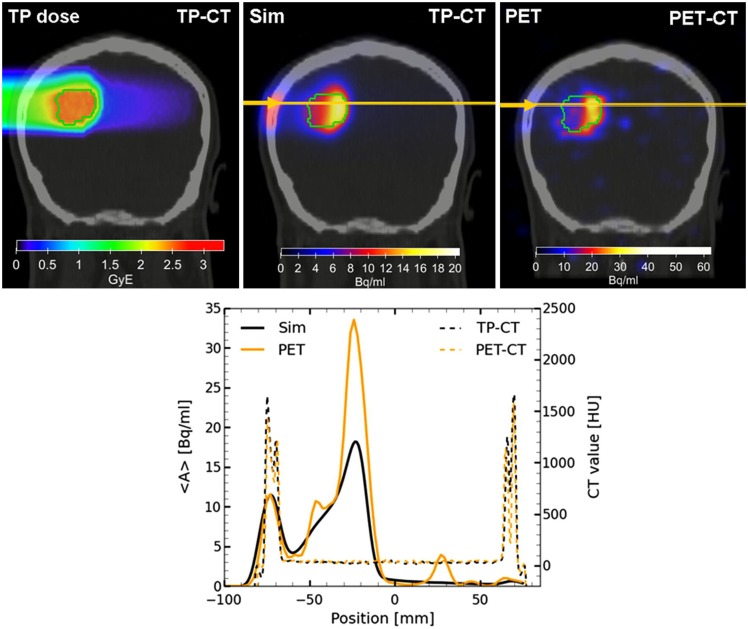
**Top left:** colourwash overlays of the planning CT image (TP-CT) in the coronal view with the RBE-weighted dose distributions as obtained from the TP system (TP dose). **Top center:** the simulated activity pattern overlaid on the PET-CT image (Sim). **Top right:** the measured PET image (PET) overlaid on the PET-CT image. The solid green line marks the planning target volume. **Bottom:** the profile plot of the simulated and the measured activity (solid) as well as the corresponding CT image values (dashed) along the yellow line in panels Sim and PET in beam direction. Reproduced from Bauer et al. (125), with permission.

Following the improvements of the internal nuclear models in FLUKA, Sommerer et al. (138) assessed the performance of FLUKA by comparing measured and simulated activity profiles in homogeneous target irradiated with carbon and oxygen beams. The code was extensively benchmarked with data and has been used for offline-treatment verification after carbon ion therapy of patients at HIT (125, 139). Figure 11 shows an example of a measured and MC predicted activity profile along the beam-axis for a glioblastoma patient treated at HIT.

Still regarding carbon treatments, Pshenichnov (70, 72, 73) has assessed the performance of the Geant4 MC code to describe PET activity measurements at GSI, using the dedicated MCHIT tool described in Section 3.2.2. However, these studies dealt mainly with homogeneous targets, and were not extended to patients.

Various groups have compared *β*^+^ activity predictions from different MC codes (101, 140, 141) with each other and with measurements, including FLUKA, Geant4/GATE, MCNPX, SHIELD-HIT, PHITS, HIBRAC, and POSGEN. These studies report large differences up to 50% in yield between the codes and the measurements, but mostly because simulations were based on different cross section models, confirming the need to use experimental cross sections in MC codes, when possible.

For other ions than protons and carbon, only few MC studies for PET-based treatment monitoring were performed. Pshenichnov et al. (73) used Geant4/MCHIT to calculate the activity induced in ^3^He treatments, and compared these predictions with data from Fiedler et al. (142), but significant discrepancies were found. PET measurements for mono-energetic ^16^O beams were presented by Sommerer et al. (138), and compared with FLUKA simulations, finding a good agreement. Finally, PET measurements with ^3^Li were performed (143) but no MC calculations were included.

Besides predictions based on full MC, various attempts to predict the PET activity analytically were done, allowing for much faster predictions. Parodi and Bortfeld (144) developed an analytical method to calculate activity by a convolution product of dose and a number of filter functions. This approach was extended by Attanasi et al. (145), but never clinically applied. Solving the inverse problem, i.e., finding the dose from activity measurements, was also studied (146, 147) with deconvolution methods, but the complexity of the problem makes it challenging to apply to real patients. Recently, Miyatake and Nishio (148) developed a promising analytical activity pencil beam algorithm.

Finally, there are treatment centers which do not use MC simulations for treatment verification. At the Kashiwa facility, treatment verification has been based on comparing the PET distributions measured during the various fractions with first day measurements (113). At the Hyogo facility, PET activity is directly compared visually with the prescribed dose to verify the beam path (149). A similar procedure but with markers was applied at the Florida Proton Therapy Institute (123).

To summarize, different approaches exist for obtaining the PET reference activity distributions. It is generally agreed on that MC predictions provide the best reference distributions. So far, the FLUKA and Geant4 generators have been used for providing reference distributions in clinical studies, yielding good agreements for PET measurements in carbon and proton irradiation.

#### 4.2.4 Challenges in Clinical Implementation

Many of the above studies have shown how PET treatment verification provides relevant clinical information. At the same time, these studies have highlighted some important limitations in the MC simulations, which should be resolved if PET is to become a widespread treatment verification technique in hadron therapy. Several issues remain to be addressed:

- Insufficient knowledge on cross section values, leading to uncertainties in *β*^+^ yield and in absolute particle range, as was shown by various studies. España et al. (133) reported that cross section uncertainties on activity fall-off position lead to a 1 and 5 mm uncertainty on the activity fall-off position, for 5 and 30 min in-room data-taking, respectively. To illustrate the problem, let’s consider Figure 6 (right), which showed the production cross section of the *β*^+^ emitter ^11^*C*. Although this plot shows large errors and conflicting data, it is an example of an accurately known cross section. In fact, for other *β*^+^ emitters (^12^N, ^14^O, ^8^B, etc), the situation is much worse, having to rely on only very few, sometimes very old, measurements including large errors to benchmark the codes. Obviously, new cross section measurements for production of various *β*^+^ emitters would be helpful. In particular, these should include systematic, high quality, double differential energy spectra, with different types of projectiles, energies, and targets.
- Inaccuracies in MC predictions from the unknown elemental composition. While for dose calculations, the CT based stoichiometric calibration is typically sufficient, this is not the case for predicting quantities heavily relying on specific nuclear reaction channels (150). The impact of CT calibrations is especially significant for proton therapy, where the *β*^+^ activity comes entirely from target residuals. The uncertainty on distal fall-off position of the PET signal was estimated to be about 1 mm for proton therapy (150). For carbon ions, the dependence is less pronounced (135). Additional information about the tissue may be extracted from the characteristic time decay curve of the PET signal (151) or from MRI.
- Inaccuracies in modeling biological washout, most relevant for off-line imaging. Our goal was to describe the physics aspects, and hence it is beyond the scope of this work to describe biological aspects. However, it has been repeatedly reported in offline PET studies that simulating biological washout correctly is highly complex and is a major limitation of the offline PET method.
- Speed and complexity of the MC simulations. The current frameworks like FLUKA and Geant4 are too slow, require a too steep learning curve, and too specialized knowledge to be applied widely in clinical practice.
- Extension of PET as monitoring tool for treatments of moving target, as for instance studied at Oncoray (137). The patient studies discussed above mostly concern static tumors. PET could be a valuable tool to monitor the accuracy of treatments of moving targets, but modeling the expected signal with MC is still largely uncovered.
- Automatic assessment of the predicted and measured activity maps, as investigated by various groups (see for instance Ref. (152, 153)).
- Translation of PET activity maps to dose. Despite the complicated and mathematically ill-defined problem, new research on this subject would be very valuable, because dose is an easily interpretable quantity for physicians. The current PET treatment monitoring is based on interpretation of activity maps, which require special knowledge about the activity signal, and is therefore much less appreciated by clinical personnel.

### 4.3 Treatment verification with prompt gammas

**Figure 12:**
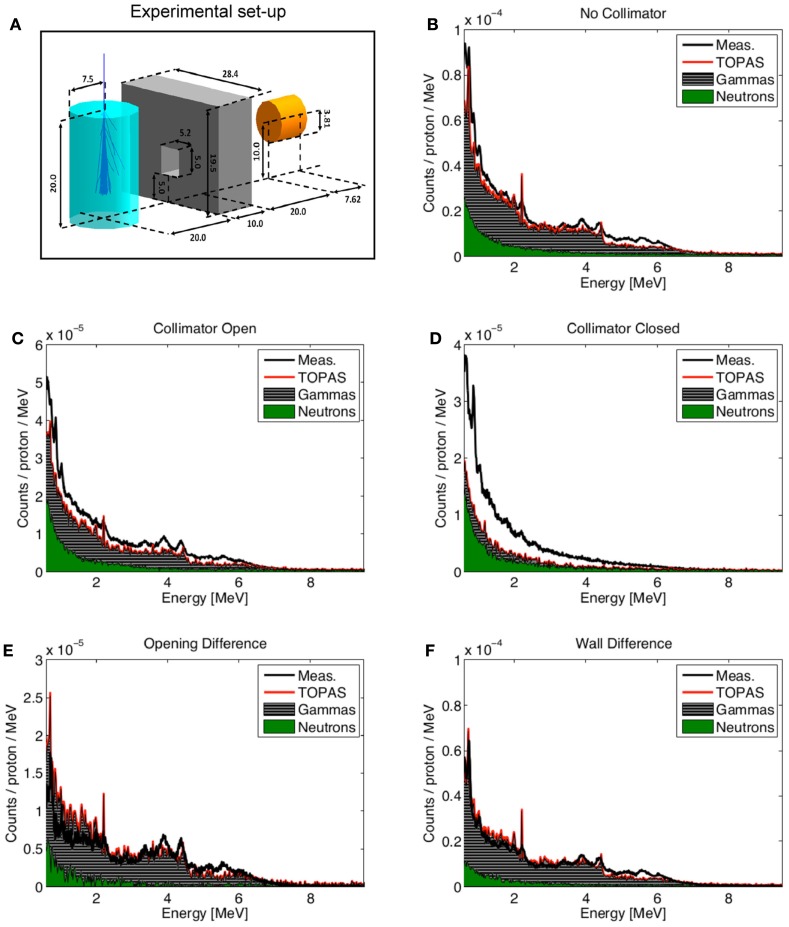
**Measured and simulated (TOPAS) energy spectra at the end of range of a monoenergetic 160 MeV proton pencil beam impinging along the central axis of a cylindrical PMMA target**. Experimental setup **(A)** together with measured and simulated energy spectra for different collimator configurations **(B–F)**. “Opening difference” is Collimator Open minus Collimator Closed, and “Wall-difference” means No Collimator minus Collimator closed, i.e., a configuration in which neutron background is subtracted. Measurements from Smeets et al. (154). Figure reproduced from Ref. (154, 155), with permission.

As discussed in Sections 2.2.2–2.2.4, prompt gammas are emitted as a result of nuclear reactions during particle delivery along much of the particle path, with energies varying from 0 to about 10 MeV (for typical spectra see Figure 12, to be discussed below). We first briefly discuss the detectors, then describe MC validation studies with prompt gammas, and finally some clinical challenges.

#### 4.3.1 Prompt Gamma Detection Devices

The energies of prompt gammas from nuclear reactions are too high for standard single gamma detection devices like SPECT to be efficient, and dedicated detector designs are needed. There are different prompt gamma imaging systems under investigation, of which some are design studies based on MC simulations, and others real prototypes. Let’s briefly discuss some of them.

- Collimated gamma cameras. By placing the camera at 90° with respect to the beam-axis and moving the device parallel to the beam-axis, a 1-D prompt gamma profile can be measured. This was the design used in the first studies where the correlation between the Bragg peak position and the prompt gamma emission profile was demonstrated, for proton (156) and carbon (157) irradiation. This design has since then been recycled by various research groups for proton therapy (158–163) and carbon treatment verification (164, 165). To increase the detection efficiency and to be able to measure the 1-D profile without having to move the detector, an array-type multi-slit camera has been designed using MCNPX simulations (166). Knife-edge-shaped slit cameras have also been investigated (154, 167, 168). Here, instead of a parallel collimator a slit-collimator is employed. Promising measurements with a collimator slit-camera prototype tested with clinical proton beams have recently been presented by Perali et al. (168), estimating a precision (*σ*) on single spot range determination of 2 mm. Recently, Pinto et al. published a review of absolute prompt gamma yields measured with proton- and carbon-ion beams with single-slit experiments (169). At phantom entrance, the average number of detected prompt gammas was found to be of order 10^−4^ per incident carbon ion and 10^−5^ per incident proton.
- Compton cameras. Here, a multi-stage detector can measure the initial energy and direction of the photons undergoing Compton scattering in the detector. Various Compton camera prototypes have been realized (170–174) and various detector design studies have been performed with Geant4 MC simulations (175–179). The advantage of the Compton camera is that it has the potential to provide 3-D images.
- Other devices. Prompt-gamma timing camera (180). This is a promising new method, where instead of the spatial position the characteristic time-of-flight of the photons is used to extract information about the particle range. The underlying idea is that the transit time from phantom entrance to detector increases with the particle range, which causes measurable effects in timing spectra. Furthermore, energy-and-time resolved prompt gamma detection has also been proposed (160, 163), and will be discussed below.

#### 4.3.2 Prompt Gamma Monte Carlo Validation Studies

A large amount of simulation and validation studies have been performed for prompt gamma imaging in proton and carbon therapy. Below we discuss some selected examples, first for proton and then for carbon therapy.

Starting with protons, Polf et al. (159) compared Geant4 simulations (version 9.1) to estimate the prompt gamma ray emission produced in water, Lucite, and bone-equivalent plastic during proton irradiation. Using a collimated gamma camera, they compared the acquired prompt gamma energy spectra with simulations, finding an overall satisfying agreement.

The MCNPX code was tested by Smeets et al. (154), who compared energy spectra measured with a knife-edge-shaped slit camera with MCNPX predictions (154). When applying a data-driven neutron background subtraction method, a satisfying agreement was obtained between data and MC simulations for the prompt gamma energy spectrum and yield. Without background subtraction, when the prompt gamma spectrum was contaminated heavily by neutron contributions, the description was unsatisfying.

Verburg et al. (181) performed an extensive validation of the nuclear cross sections of specific gamma-emission channels, identified by lines in the measured energy spectra, of the Geant4 (9.5) and MCNP6 codes, as well as for two pre-equilibrium reaction codes (TALYS and EMPIRE), for protons up to 200 MeV. Cross section predictions as a function of incident proton energy of the MC codes were compared to evaluated data from the ENDF/B-VII database. Using the BIC model for Geant4 and the Bertini model in MCNP6, significant differences were found between measurements and predictions of the most important reaction channels, mostly in the low energy region (<20 MeV) where the codes tend to underestimate the cross sections by a factor two. The TALYS and EMPIRE values were somewhat better.

Still focusing on protons, a recent study by Dedes et al. (182) investigated the accuracy of Geant4 code (version 9.4) using the BIC model for nucleon-nucleus interactions. Different measurements of prompt gamma energy spectra with a collimated camera placed at different angles (mostly 90° w.r.t. the beam axis) were performed, and compared to Geant4 predictions. Additionally, the measurements from Smeets et al. (154) were used for comparison. The prompt gamma yield was generally overestimated using the Geant4 BIC model, evidencing the need for further improvements in the nuclear models.

A similar study was recently performed with TOPAS (155), where the same measured energy spectra from Smeets et al. (154) were compared with TOPAS simulations, also using the Geant4 BIC model. In contrast to Dedes et al. (182), an overall good agreement in yield and prompt gamma spectra between TOPAS predictions and measurements were obtained, when subtracting the neutron background. The results are shown in Figure 12. Moreover, the accuracy of prompt gamma imaging was estimated for a clinical scenario. A 4 mm accuracy was estimated for a prostate tumor treatment with a dose of only 15 cGy delivered with passively scattered protons, a promising result.

Closely related to range monitoring, research has been performed to evaluate the sensitivity of the prompt gamma energy spectra in proton therapy to tissue composition (160, 163, 183). At the end of range, when the projectile energy has decreased and only a few relaxation channels are possible, discrete lines in the gamma spectrum are visible (see Figure 12). These have been shown to be sensitive to the elemental composition of the sample (160, 183). In particular, the measured spectra can be used directly as input in the MC predictions to increase their accuracy (163).

For carbon beams, the Geant4 performance for prompt gamma predictions was tested in several studies using the older nuclear INC models (184) and newly implemented QMD model (182). At low energies (95 MeV/u), the QMD model describes well energy spectra and yields when tuning the free parameters in the model. At higher energies, the observed remaining overestimation by Geant4 comes from the secondary proton and neutron contributions, which are not correctly described by the BIC model, as was seen also in the previously mentioned study by Dedes et al. (182).

**Figure 13:**
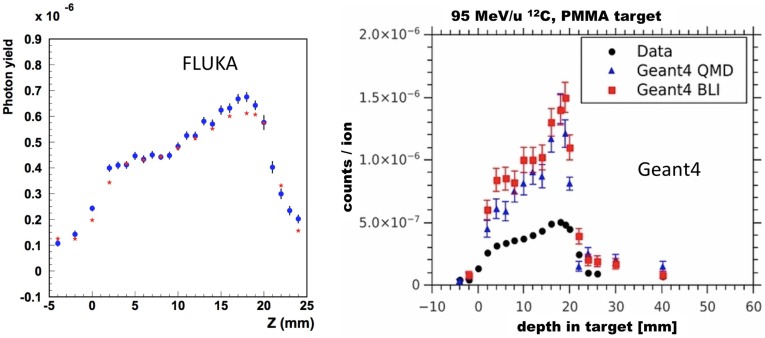
**Left:** prompt photon yield at 90° as a function of depth for a 95 MeV/n ^12^C beam impinging on a PMMA target. The Bragg peak position is at about 20 mm. Data (red stars) are from Ref. (184), re-evaluated as described in Ref. (169), and FLUKA simulations (blue circles) are shown. Reproduced from Ref. (55), with permission. **Right:** the same data, compared with simulations of Geant4 using the QMD model with different values of a free parameter. Reproduced from Ref. (182), with permission.

Recently, the performance of FLUKA was investigated for prompt gamma production in ^12^C irradiation of a PMMA target (55). An example of a predicted spatial prompt gamma profile along the beam path measured at 90°(from Ref. (184)) is given in Figure 13, showing a good agreement.

Finally, there are studies exploiting a completely different type of prompt gammas, coming from Cherenkov radiation from secondary electrons produced during particle irradiation (185). In this context, a study by Yamaguchi et al. (186) measured low energy prompt photons (around 65 keV) to verify the ^12^C range, which could provide a complementary approach to other methods for range verification for shallow target treatments.

To summarize, for proton beams, a reasonable description of the prompt gamma yield could be obtained, although disagreements were reported as well, especially to describe neutron contributions. Concerning carbon beams, QMD models are generally still in a development status, but current implementations are promising.

#### 4.3.3 Challenges for Clinical Implementation

The clinical implementation of the prompt gamma technique is still facing several challenges, which are discussed elsewhere (4). Concerning MC modeling, we have seen that the accuracy of the MC simulations has been much improved recently, but many issues still remain to be improved. Eventually, the prompt gamma imaging method will depend on comparisons between data and expectations calculated from 3-D patient descriptions like CT’s, just like the PET imaging method. Whichever measurements will be performed (1-D, 2-D, 3-D spatial distribution, timing profiles, energy spectra), the MC codes will provide the reference. Current challenges in signal modeling to be addressed include:

- Nuclear models in most MC generators are not fully satisfying. Predicted cross sections are not always in agreement with data, and the neutron background is poorly understood. New cross section measurements at various angles for protons and carbon beams on thin targets covering the whole range of therapeutic energies would be useful (160, 163).
- Increasing the speed of the calculations is also important. Research on how to perform faster and more efficient calculations of prompt gamma yields was presented in recent articles (104, 187). Separating the MC physics generation step and the detector simulation step as done in Ref. (166) can additionally help.
- In-house validation of the applied MC codes. Each research center has to validate its own MC framework using their own beamline and detection system, as was shown to work best also for PET monitoring. MC validation tests with the help of the standard QA measurements as proposed in Ref. (155) should be a starting point for validating the code.
- Performing data-MC comparisons for heterogeneous targets, including patients. Although studies on heterogeneous phantoms have been initiated (188), much more research is needed to verify the reliability of the MC codes when the phantom is heterogeneous and when the elemental composition must be obtained from a CT scan.
- Performing uncertainty analysis, for instance quantifying the influence of CT calibrations, ionization potential, or nuclear cross sections on the MC predictions, as has been studied for PET imaging too.

### 4.4 Charged particle imaging

Another method which offers the possibility to determine the particle range is the analysis of the charged particles that are created during nuclear fragmentation in the patient (Section 2.2), and which exit the patient (189, 190). This method has so far been limited to carbon irradiation, where the amount of high-energy secondary charged particles is larger than in proton therapy.

In the interaction vertex imaging (IVI) method, the trajectories of the charged particles exiting from a target are reconstructed and extrapolated back to their production point. Henriquet et al. (191) presented a feasibility study for this technique in carbon therapy, using Geant4 (9.2) simulations. An angle of 30° with respect to the beam-axis was chosen to detect charged particles. For homogeneous phantoms, milimetric precision was expected when monitoring with single pencil beams of 2 × 10^5^ carbon ions. The approach was experimentally tested by Gwosch et al. (192), measuring charged particles exciting from homogeneous targets irradiated with carbon ions at HIT. The tracking device was placed at an angle of 30° from the beam-axis. The accuracy for monitoring the beam-range was found to be 1–3 mm, but based on pencil beams with much higher statistics than what is used clinically.

**Figure 14:**
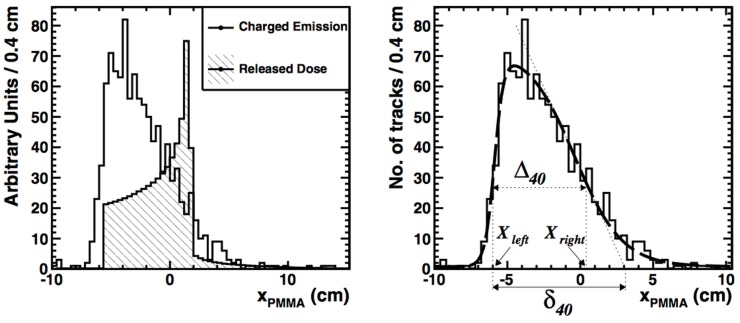
**Left:** FLUKA simulation of the depth-dose profile (hatched histogram) superimposed on the longitudinal profile of charged secondary particles (solid line) as a function of *x_PMMA_*, the primary beam direction. **Right:** the corresponding data (histogram) and data analysis (smooth line). Reproduced from Ref. (194), with permission.

Detection of secondary charged particles for range monitoring was also investigated by others. Agodi et al. (193) and Piersanti et al. (194) irradiated a PMMA target with mono-energetic carbon ions with various energies. Trajectories of charged particles with kinetic energies up to several tens of MeV were measured with a tracking device, placed at 60° and 90° angle with respect to the beam axis. A clear correlation between the measured 1-D profile of the charged particle yield and the dose was found, and a reasonable agreement with FLUKA predictions (see Figure 14). These measurements at large angle are very valuable for the validation of nuclear models in MC codes.

A large-area proton range telescope is being developed by the TERA collaboration (195), with expected acceptance of 30 cm× 30 cm perpendicular to the beam.

Current challenges in signal modeling being faced for this range monitoring technique are very similar to those already mentioned in PET and prompt gamma monitoring. However, a very accurate MC prediction of the angular distributions of the fragments is even more crucial in this case, relying on prediction at large angles. Double differential energy spectra are especially useful for the validation of MC codes, such as recent measurements by De Napoli et al. (76) and Dudouet et al. (77). Concerning the detector, the acceptance and efficiency should still be increased.

## 5 Discussion and Future Outlook

The enormous amount of literature written about range monitoring demonstrates the worldwide interest in the subject. With the number of particle facilities growing, and in view of the increasingly considered hypofractionation schemes for dose delivery, non-invasive particle range verification methods will become even more needed in the future. MC simulations are of prime importance in the development and application of range monitoring. In this review, we intended to describe the physics modeling and MC codes that are applied in the currently most widely researched range monitoring techniques, and to highlight therein the difficulties and challenges.

We have seen that the main inaccuracies in physics modeling have turned out to be very similar for the three techniques, because they rely all on an accurate description of electromagnetic and nuclear interactions of hadrons in matter. Summarizing, the common inaccuracies include:

- Uncertainties in describing nuclear interactions, including cross sections and interaction models. For PET, we saw in Section 4.2.4 that cross section uncertainties can lead to uncertainties in activity distal fall-off position of several millimeters. New double differential thin and thick target cross section measurements for specific reaction products in the entire energy range of therapeutic protons and heavy ions are the best way to improve the accuracy.
- Uncertainties in densities and elemental composition of the target. For PET, such uncertainties were of the order of a millimeter for proton therapy, as mentioned in Section 4.2.4. MRI could possibly improve the knowledge on elemental composition, as well as evaluating the elemental composition during range monitoring. For PET-based methods, the latter can be done by measuring time-decay curves of the PET signal, as was remarked in Section 4.2.4, while for prompt *γ* monitoring the discrete spectral lines could be used, as referenced in Section 4.3.2.
- Uncertainties on the position of the Bragg peak, coming from uncertainties in the ionization potential *I* and in the ionization model. As was discussed in Section 2.4, variations in the measured value of *I* in water lead to an uncertainty of several percent in Bragg peak position. In-house Bragg peak measurements could help to validate the MC code, thereby reducing this uncertainty.

For PET treatment verification, these uncertainties can add up to several millimeters, with nuclear interaction modeling as main source of uncertainty. For prompt gammas and charged particle imaging, the uncertainties have not been quantified, but are probably of similar size. A clinically valuable system should ideally provide a 1–2 mm estimation on range, preferably using single or few spots in the treatment plan, i.e., 10^8^ and 10^6^ particles for proton and carbon therapy. Thus, reducing the MC uncertainties to below 1–2 mm is crucial. However, it could be a shared effort for the PET, prompt gamma, and charged particle imaging communities.

Keeping in mind the underlying physics and the achieved results of the three monitoring techniques, let’s briefly summarize their advantages and disadvantages. Starting with PET, this is a well-established method proven to provide clinically useful post-treatment information on the dose delivery. Unfortunately, the response-time is intrinsically limited by the decay time of the *β*^+^ emitters. However, with online PET systems that acquire data during irradiation, a relatively quick response is expected, making such systems particularly valuable. Geometrical problems in planar configurations can be decreased when TOF information is used or with innovative geometrical designs. So far, post-treatment verification has been performed based on entire treatment plans, so that enough statistics is collected. Pre-treatment range measurements with one or a few single pencil-beams are difficult, but using larger online detectors it may be feasible in the future.

Prompt gamma detection has an important advantage with respect to PET, because prompt gammas are produced immediately when irradiating a target. This technique can thus provide real-time information, and issues with biological washout or movement are absent. Moreover the number of prompt gamma events produced is much larger than the number of annihilation photons used in PET treatment verification. However, much research in detector development is still needed to bring this technique to the clinic. Single pencil-beam monitoring seems feasible, although additional research is needed to confirm this. The accuracy of MC simulations has been much improved recently, but some crucial issues still remain to be studied, including neutron backgrounds, as well as dedicated studies with heterogeneous phantoms and patients.

Charged particle measurements can additionally provide a way to monitor the range. Predictions of MC codes for secondary particle production at large angles are generally not yet fully satisfactory. Although the expected sensitivity is smaller than with PET and prompt gammas, charged particles could for instance provide useful additional information in combination with other techniques, i.e., as part of a “hybrid” system. An example of such a system is being built in the framework of the INSIDE project (196), where a planar TOF PET system is combined with a tracking system to provide range monitoring measurements at the CNAO treatment facility in Pavia, Italy. More MC studies to assess the value of this kind of hybrid systems would be very useful, for instance it would be highly interesting to study triple system, combining PET, prompt gamma, and charged particle measurements.

Direct comparison studies with MC simulations are a good way to compare the techniques. However, such studies are scarce. Moteabbed et al. (197) performed a patient simulation study with Geant4 comparing the PET and prompt gamma techniques in proton treatments. They found that prompt gamma imaging was potentially advantageous for certain tumor types; however, the study was based on in-room PET and moreover the Geant4 code has significantly changed. New comparison studies between the various techniques would therefore be timely. Since the accuracy of each technique undoubtedly depends on treatment site, tumor type, depth, volume, treatment plan, particle beam, and so on, it is important that such comparison studies include large patient groups, and present their results as quantitative as possible.

Finally, the diversity of the literature studied here, encompassing nuclear physics, MC codes, detectors, and clinical challenges, highlights how much knowledge from different fields has been combined in the developments of range monitoring strategies. In particular, the literature studied to describe the modeling of nuclear interactions in the human body covers a time span of more than 70 years. Having even omitted biological issues, it is clear that modeling the underlying physics in MC codes and developing the ultimate range verification technique requires expertise which goes far beyond the field of medical physics alone.

## 6 Conclusion

This review was aimed at providing a description of the most relevant aspects of the underlying physics and modeling in MC codes used in treatment monitoring techniques based on secondary particle detection. The complexity and variety of the underlying physics makes an accurate description of the production of secondary particles a highly challenging and non-trivial task. We have shown how various research groups validate and apply different MC codes to obtain their reference distributions, needed for a comparison with data.

## Conflict of Interest Statement

The author declares that the research was conducted in the absence of any commercial or financial relationships that could be construed as a potential conflict of interest.

## Footnotes

^1^The *intranuclear* cascade refers to the cascade inside the nucleus, as opposed to the *inter-nuclear* transport of a particle from one nucleus to another.
